# Circulating tumor cells (CTC) and KRAS mutant circulating free DNA (cfDNA) detection in peripheral blood as biomarkers in patients diagnosed with exocrine pancreatic cancer

**DOI:** 10.1186/s12885-015-1779-7

**Published:** 2015-10-24

**Authors:** Julie Earl, Sandra Garcia-Nieto, Jose Carlos Martinez-Avila, José Montans, Alfonso Sanjuanbenito, Mercedes Rodríguez-Garrote, Eduardo Lisa, Elena Mendía, Eduardo Lobo, Núria Malats, Alfredo Carrato, Carmen Guillen-Ponce

**Affiliations:** 1 Medical Oncology Department, Ramón y Cajal University Hospital, Carretera de Colmenar Viejo, KM 9,100, 28034 Madrid, Spain; 2 Genetic and Molecular Epidemiology Group, Spanish Cancer Research Cancer Center, Madrid, Spain; 3 Pathology Department, Ramón y Cajal University Hospital, Madrid, Spain; 4 Surgery Department, Ramón y Cajal University Hospital, Madrid, Spain

**Keywords:** Circulating Free DNA, KRAS mutation, Circulating Tumor Cells, PDAC, Prognostic Marker

## Abstract

**Background:**

Pancreatic cancer remains one of the most difficult cancers to treat with the poorest prognosis. The key to improving survival rates in this disease is early detection and monitoring of disseminated and residual disease. However, this is hindered due to lack reliable diagnostic and predictive markers which mean that the majority of patients succumb to their condition within a few months.

**Methods:**

We present a pilot study of the detection circulating free DNA (cfDNA) combined with tumor specific mutation detection by digital PCR as a novel minimally invasive biomarker in pancreatic ductal adenocarcinoma (PDAC). This was compared to the detection of CTC by the CellSearch® system and a novel CTC enrichment strategy based on CD45 positive cell depletion. The aim of the study was to assess tumor specific DNA detection in plasma and CTC detection as prognostic markers in PDAC.

**Results:**

We detected KRAS mutant cfDNA in 26 % of patients of all stages and this correlated strongly with Overall Survival (OS), 60 days (95 % CI: 19–317) for KRAS mutation positive vs 772 days for KRAS mutation negative (95 % CI: 416–1127). Although, the presence of CTC detected by the CellSearch® system did correlate significantly with OS, 88 days (95 % CI: 27–206) CTC positive vs 393 days CTC negative (95 % CI: 284–501), CTC were detected in only 20 % of patients, the majority of which had metastatic disease, whereas KRAS mutant cfDNA was detected in patients with both resectable and advanced disease.

**Conclusions:**

Tumor specific cfDNA detection and CTC detection are promising markers for the management of patients with PDAC, although there is a need to validate these results in a larger patient cohort and optimize the detection of CTC in PDAC by applying the appropriate markers for their detection.

**Electronic supplementary material:**

The online version of this article (doi:10.1186/s12885-015-1779-7) contains supplementary material, which is available to authorized users.

## Background

Pancreatic ductal adenocarcinoma (PDAC) is the most common cancer affecting the exocrine pancreas. In Europe there are 60,139 new diagnoses and 64,801 deaths very year [1]. The prognosis of patients is dismal with a 5 year survival rate of around 5 % as the majority of patients diagnosed with PDAC present with an advanced disease and distant metastasis. Surgical resection of the primary tumor is the only hope for a cure but unfortunately this is only possible in around 15–20 % of patients.

There have been considerable improvements in long-term survival following PDAC resection over last few decades with 5-year survival rates of approximately 27 % [2], however, 80 % of patients relapse within months after an attempt at curative surgery [3]. There are several prognostic factors and predictors of relapse such as tumor aneuploidy, positive lymph nodes, tumor size, poor histological tumor differentiation and positive resection margins but there is a need for additional accurate and reliable markers for effective monitoring of disease evolution with regard to disease dissemination in localized tumors and residual disease after treatment in advanced patients.

The most commonly used tumor biomarker in PDAC is carbohydrate antigen 19–9 (CA 19–9), the sensitivity is around 79 % and specificity 82 %. However, CA19-9 levels increase in other non-malignant pancreatic disorders such as acute pancreatitis and other gastrointestinal malignancies [4, 5]. Circulating branched-chain amino acids have also been proposed as a novel biomarker appearing 2–5 years before diagnosis [6]. However, there is still a need for new diagnostic and predictive biomarkers that complement imaging techniques used in patient follow-up in order to achieve a more effective management of these patients and improve survival.

The presence of circulating tumor cells (CTC) in peripheral blood has been associated with a reduced progression free survival (PFS) and overall survival (OS) in some cancer types and may be useful as an early indicator of tumor spread, as invasive but localized tumors may shed CTC into the blood stream before a metastasis is established. The CellSearch® system enumerates CTC based on the expression of epithelial markers and has been used extensively in predicting prognosis and response to treatment in breast, colon, lung and prostate cancers [7–10] although there are few studies of CTC as a biomarker in PDAC. 45 % of patients with stage IV disease tested positive for CTC in one study whereas 5 % of patients with a locally advanced disease were CTC positive in another study using the CellSearch® system [11, 12]. A comparative study in metastatic or inoperable pancreatic cancer detected CTC in 40 % of patients using the CellSearch® system as compared to 93 % by ISET (Isolation by Size of Tumor cells), on the whole more CTCS were detected by ISET than by CellSearch®, mean 26 versus 2 CTCs/7.5 ml of blood (range 0–240 versus 0–15) [13]. The limitation of the cell search system is that circulating tumor cells that do not express the marker EpCAM and/or Cytokeratins 8, 18 and 19 will not be detected by the system. Other CTC detection systems include the isoflux, ImageStream^X^systems, however, these have not been validated in the context of pancreatic cancer.

Nucleic acids are released and circulate in the peripheral due to apoptosis and necrosis of cells. During tumorigenesis there is an increase in cell turnover and thus more cell necrosis and apoptosis which is released into the blood stream and leads to an accumulation of cfDNA, thus cancer patients tend to have more cfDNA than non-cancer patients [14]. Thus, cfDNA has been exploited as a cancer biomarker, high plasma cfDNA content is associated with poor survival in patients with lung adenocarcinoma, similarly a study in colorectal cancer has shown that the concentration of cfDNA correlates strongly with clinical outcome [15, 16]. One drawback of this approach is that cfDNA content may increase in non-cancer states such as benign tumors and inflammatory diseases thus DNA concentration alone is not an adequate marker to distinguish between cancer and non-cancer states. Thus it would be ideal to use this in combination with tumor specific DNA mutation detection, such as mutant KRAS, which is the most common genetic alteration found in PDAC occurring in approximately 90 % of tumors [17].

This is an exploratory study of tumor specific mutation detection in cfDNA in patients diagnosed with PDAC. In addition, we evaluate the quantification of cfDNA in plasma, tumor specific mutation detection in plasma as well as CTC detection in peripheral blood as prognostic biomarkers in PDAC using overall survival analysis.

## Methods

### Patients

Patients were recruited via the Medical Oncology and Surgery Departments at the Ramón y Cajal hospital, Madrid, Spain between October 2009 and May 2014. The study was approved by the clinical investigation ethics committee of the Ramón y Cajal University Hospital and all participants signed the associated informed consent form. The study included a total of 45 patients with histological or cytological confirmed PDAC diagnosed at different disease stages (resectable, locally advanced and metastatic disease). The patients were divided into 2 cohorts; this included (1) 31 patients with cfDNA concentration and KRAS mutation detection data and (2) 35 patients with CTC data. 21 patients had both sets of data. When possible, samples were taken prior to starting treatment, either surgery or chemotherapy, although 7 patients had previously received gemcitabine chemotherapy before the sample was taken.

### cfDNA detection and quantification by digital PCR

cfDNA was extracted from 1 ml of plasma using the QIAamp Circulating nucleic acid kit (Qiagen), DNA was isolated in a final volume of 50 μl. The total DNA concentration in plasma was estimated by determination of the number of copies of the RNaseP (RPP30) gene, as this gene is rarely affected by mutations or copy number alterations. The number of copies of the RNaseP gene was determined by ddPCR amplification using the QX200™ Droplet Digital™ PCR System (BioRad) using a specific PrimePCR copy number assay (BioRad, RPP30 dHsaCP1000485) according to the manufacturer’s instructions. 1 μl of isolated cfDNA corresponding to 20 μl of plasma was used as a template for each PCR and reactions were performed in duplicate with non-template negative controls.

Absolute quantities of RNaseP DNA copies were determined using the QuantaSoft software supplied by the manufacturer. Briefly, a fluorescence intensity threshold of 3000 was set and all droplets above this threshold were scored as positive. Each positive droplet corresponded to a single copy of the RNaseP gene. cfDNA concentration was expressed as the total number of copies of RNaseP in 20 μl of plasma.

### Tumor specific mutation detection in cfDNA by digital PCR

Information on the frequency of mutations in KRAS in primary PDAC was retrieved from the COSMIC database [17]. The QX200TM Droplet Digital PCR System (Biorad) and the PrimePCR KRAS mutant assays (Biorad, dHsaCP2000001 (G12D), dHsaCP2000009 (G12R), dHsaCP2000005 (G12V),) and corresponding WT assays (dHsaCP2000002 (G12D), dHsaCP2000006 (G12V), dHsaCP2000010 (G12R)) were used to detect the following KRAS mutations in cfDNA: G12D, G12R and G12V. 1 μl of isolated cfDNA was used as a template for each PCR. Duplicates samples were analyzed as well as the corresponding mutation positive control DNA for the mutations tested. The positive control DNA for each assay was also used as a negative control for other assays in order to determine the level of non-specific amplification. Additional non-template negative controls were also included.

Following PCR amplification, absolute quantities of mutant and WT DNA copies were determined using the QuantaSoft software as previously described. Briefly, the system uses a 2 color detection system for the WT (FAM) and Mutant (HEX) alleles to count the number of droplets positive for each fluorophore. We considered samples as positive for mutant KRAS when at least 3 positive HEX droplets were identified above the threshold level.

### KRAS mutation detection by ddPCR in plasma spiked with KRAS mutant DNA

1 ml of plasma from a healthy control was spiked with 250 ng, 100 ng, 50 ng and 25 ng of DNA from the pancreas cancer cell line, SUIT-2, that harbors the G12D KRAS mutation. cfDNA was extracted from these samples as well 1 ml of un-spiked plasma and G12D KRAS mutation detection by ddPCR was performed as previously described.

### Genomic DNA extraction and KRAS sequencing in primary tumors

Paraffin embedded tissue from primary tumors was assessed by an experienced pathologist and an area corresponding to tumor was selected for DNA extraction. The tumor content was macro dissected by tissue punch. Genomic DNA was extracted from 12 paraffin embedded primary tumor tissue using the Qiagen DNeasy Blood and Tissue kit and exon 2 and 3 of the KRAS gene was amplified using the following primers KRAS exon 2 fwd 5′ACACGTCTGCAGTCAACTGG-3′ KRAS exon 2 rev 5′-TAACTTGAAACCCAAGGTAC-3, KRAS exon 3 fwd 5′-GCACTGTAATAATCCAGACT-3 KRAS exon 3 rev 5′-CATGGCATTAGCAAAGACTC-3. The products were sequenced by Sanger sequencing using the Big Dye® Terminator v3.1 cycle sequencing kit (ABI) according to the manufacturer’s instructions in order to verify the presence of a KRAS mutation.

### CTC determination by CellSearch®

Briefly, 7.5 ml of blood was mixed with sample buffer and centrifuged before loading into the CellSearch® (Janssen) instrument for subsequent automated processing. The CellSearch® system contains a ferro fluid-based capture reagent targeting the EpCAM antigen of CTC and immunofluorescent reagents targeting the intracellular protein cytokeratin (epithelial cells), DAPI (nucleus) and CD45 (leukocytes) for the identification and enumeration of CTC. The Celltracks Analyzer II^®^ System scans samples and identifies events where cytokeratin and DAPI fluorescence are co-located. An event is classified as a tumor cell when complying with the following criteria; (1) Morphology: a round or oval intact cell with a minimum size of 4 microns (2) EpCAM positive, cytokeratin positive, DAPI positive and CD45 negative (3) At least 50 % of the nucleus must be visible inside the cytoplasm. A CellSearch® Circulating Tumor Cell Control was analyzed in each sample run which checks the overall system performance, including the instrument, reagents and operator technique.

7.5 ml of peripheral blood was spiked with 750 cells of the human pancreatic cancer cell lines AsPc-1 and PaTu899S to obtain 100 cells per ml of blood; these acted as pancreatic cancer tumor cell positive controls and were processed as described previously. CTC calling was performed by trained personnel and verified by an independent expert. According to the manufacturer, the mean CTC count in healthy individuals is 0.1 (*N =* 145, SD = 0.2) and 0.1 (*N =* 99, SD = 0.4) in patients with non-malignant disease. We classified a sample as positive when 1 CTC was detected.

### Enrichment of CTC by CD45 positive cell depletion in peripheral blood

4 ml of blood was used to isolate and enrich circulating tumor cells. Red blood cells were lysed using a hypotonic solution of ammonium chloride. Magnetic Activated Cell Sorting (MACS) was used to remove haematopoietic cells that express the cell surface marker CD45 as described by the manufacturer. Briefly, cells were counted after red blood cell lysis and cells were resuspended in 80 μl of MACS buffer (PBS + 0.5 % BSA + 2 mM EDTA) with 20 μl of magnetically labelled CD45 antibody per 1 million cells. After incubation at 4 °C for 15 min the cells were washed twice in MACS buffer and CD45 positive and negative cells were separated using MACS ferromagnetic columns and washed in PBS before DNA extraction.

### Genomic DNA extraction and KRAS sequencing in CD45 positive cell depleted blood

DNA was extracted from 9 CD45 negative isolated cell population specimens using the Qiagen DNeasy Blood and Tissue kit and exon 2 and 3 of the KRAS gene were PCR amplified and sequenced as previously described.

### Statistical analysis

Statistical Analysis was performed using R [18] and SPSS [19]. Differences in age for the patient cohorts with available data for CTC determination and KRAS mutation in cfDNA were assessed with the non parametric Mann–Whitney test. The Fisher exact test was applied for the categorical variables such as sex and stage. The Mann–Whitney was used to assess the differences in concentration of cfDNA across the 3 disease stage groups (resectable, locally advanced and metastatic), as well as the assessment of differences in cfDNA concentration according to KRAS mutation status. The Pearson correlation was applied to determine the correlation between KRAS G12D DNA spike in concentration and the number of G12D copies detected by ddPCR. Survival analysis with regard to CTC and KRAS mutation detection in cfDNA was assessed in three ways. First, a univariate analysis was performed using the Kaplan Meier estimate of survival to compare CTC or mutant KRAS positive vs negative patients with the Mantel-Haenszel test. Second a Cox regression was fitted that included sex and age as confounders. Finally a Weibull regression analysis was performed using the parameters sex and age.

## Results

### Patient characteristics

The characteristics of the 45 patients included in the study are shown in Table 1. 24 patients were male and 21 female, the median age at diagnosis was 68 years of age (66 years of age for males and 69.5 years of age for females). Patients were divided into 3 clinical groups: (1) patients with a localized that are eligible for surgical resection (R), (2) patients with a locally advanced disease but not eligible for surgery (LA), (3) patients with stage IV metastatic disease (M). Tweenty-one patients had both sets of data. Statistical analysis of the cohorts of patients with cfDNA data, CTC data or both data showed that they were equivalent populations in terms of sex and stage, although the cfDNA only group had a younger age at diagnosis (Additional file 1: Table S1).

**Table 1:** Characteristics of the PDAC patients included in the study

Patient Code	Disease Stage	QT before CTC/KRAS cfDNA determination	KRAS cfDNA data	CTC data	CTC/KRAS cfDNA data	DNA concentration in plamsa (Average copies RNaseP/20ul plasma)	KRAS status in plasma	KRAS Mutation in plasma	Ratio M:WT KRAS in plasma	CTC STATUS	Number of CTC	CD45 Depletion KRAS mutation	Mutation in Tissue
1	R	YES	YES			80	NEG						G12D
2	R	YES	YES			43	NEG						G12R
3	R	YES	YES			59	NEG						
4	R	NO	YES	YES	YES	106	NEG			NEG	0		
5	R	NO	YES	YES	YES	97	NEG			NEG	0	WT	WT
6	R	NO	YES	YES	YES	185	POS	G12D	0,21	POS	1		G12D
7	R	NO		YES						NEG	0	WT	
8	R	NO		YES						NEG	0		
9	R	NO	YES			86	POS	G12D	0,1			WT	WT
10	R	NO	YES	YES	YES	93	POS	G12D	0,01	NEG	0		G12D
11	R	NO	YES	YES	YES	48	NEG			NEG	0		G12S
12	R	NO	YES	YES	YES	1541	NEG			NEG	0		
13	R	NO		YES						NEG	0		
14	R	NO		YES						NEG	0		
15	LA	NO	YES			52	POS	G12V	0,12				
16	LA	NO	YES	YES	yes	6,4	NEG			NEG	0		
17	LA	NO	YES	YES	YES	66	NEG			NEG	0	G12D	
18	LA	NO	YES	YES	YES	1063	NEG			NEG	0		
19	LA	NO		YES						NEG	0		
20	LA	YES	YES			297	NEG						
21	LA	NO	YES	YES	YES	700	NEG			NEG	0		
22	LA	YES	YES	YES	YES	38	NEG			NEG	0		
23	LA	NO	YES	YES	YES	111	NEG			NEG	0		
24	LA	NO		YES						NEG	0		
25	LA	NO		YES						NEG	0	G12D	
26	LA	NO		YES						NEG	0		
27	LA	NO		YES						NEG	0		
28	M	NO	YES			806	POS	G12D	0,06				
29	M	NO	YES	YES	YES	12,2	NEG			NEG	0		
30	M	YES	YES	YES	YES	1663	POS	G12D	2,43	POS	5		WT
31	M	NO	YES	YES	YES	72	NEG			NEG	0		WT
32	M	NO	YES	YES	YES	1095	POS	G12R	0,02	POS	4		G12R
33	M	NO		YES						NEG	0		
34	M	NO		YES						NEG	0		
35	M	NO		YES						POS	3		
36	M	NO	YES			130	NEG						
37	M	NO	YES			147	NEG					G12D	
38	M	YES	YES			87	NEG						G12D
39	M	NO	YES	YES	YES	33	NEG			NEG	0		WT
40	M	NO	YES	YES	YES	328	NEG			NEG	0	G12D	
41	M	NO	YES	YES	YES	602	POS	G12D	0,81	POS	3		
42	M	NO	YES	YES	YES	70	NEG			NEG	0	WT	
43	M	NO	YES	YES	YES	52	NEG			POS	1	WT	
44	M	NO		YES						NEG	0		
45	M	NO		YES						POS	13		

*R* Resectable, *LA* Locally Advanced, *M* Metastatic

### Measurement of DNA concentration in plasma

**Fig. 1:**
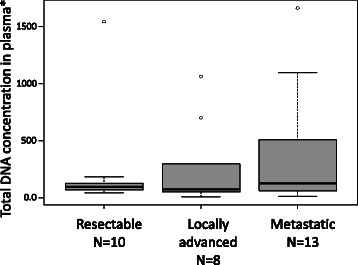
Correlation of total cfDNA concentration in plasma with PDAC disease stage. *DNA concentration was estimated by the number of copies of the RNaseP gene in 20 μl of cfDNA in plasma

The number of copies of the RNaseP gene was taken as a measurement of total DNA concentration in plasma samples. This information was available for 31 patients (Table 1). The median number of copies of the RNaseP gene in 20 μl of plasma was 93 (range 6–1663, 25 % percentile 55.5 and 75 % percentile 312.5). DNA concentration in plasma tended to increase with increasing disease stage although this correlation did not reach statistical significance (Fig. 1). There was no obvious correlation with OS based only on DNA concentration in plasma.

### Specificity of KRAS ddPCR mutation assays

The specificity of the G12D, G12R and G12V KRAS mutation assays was tested by ddPCR amplification of DNA samples harboring these 3 mutations. The results are shown in Additional file 2: Figure S1. There was no non-specific amplification above the threshold level with the G12D and G12R assays. However, there was non-specific amplification of G12D mutant DNA with the G12V assay.

### KRAS mutation detection in spiked plasma by ddPCR

Plasma spiked with KRAS G12D mutant DNA and analyzed by ddPCR is shown in Additional file 3: Figure S2a. The number of G12D mutant copies detected in each spike in plasma is shown in Additional file 3: Figure S2b. The correlation coefficient between the number of G12D copies detected by ddPCR and the spike in concentration was 0.99 (*p <* 0.01). The system detected KRAS G12D mutant spike in DNA down to a concentration of 0.5 ng which represented 37 mutant copies.

### KRAS detection in cfDNA using digital PCR

**Fig. 2:**
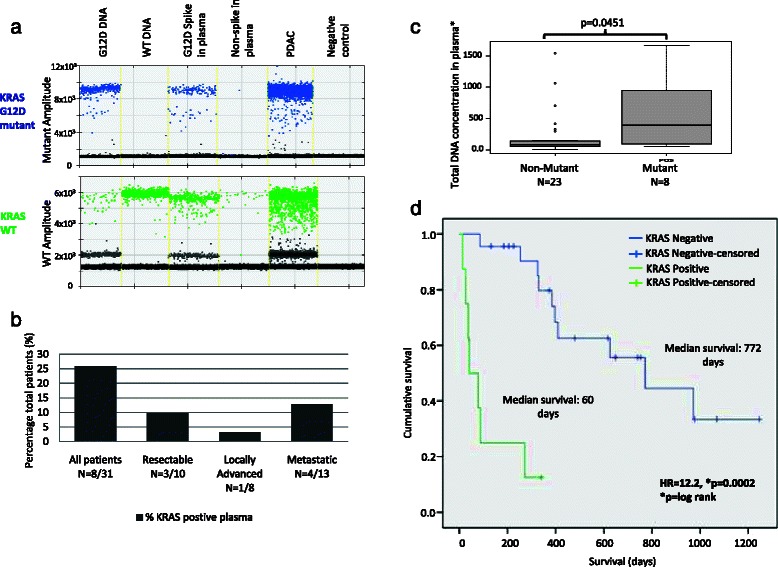
KRAS mutation detection in plasma cfDNA in PDAC cases. **a**. G12D KRAS mutation detection in plasma and genomic DNA by QX200™ Droplet Digital™ PCR. **b**. Frequency of mutant KRAS detection in plasma in PDAC. **c**. Correlation of cfDNA concentration and mutant KRAS detection. *DNA concentration was estimated by the number of copies of the RNaseP gene in 20 μl of cfDNA in plasma. **d**. Kaplan Meier survival analysis of KRAS mutation status in plasma cfDNA

KRAS mutation detection in cfDNA data for the mutations G12D, G12V and G12R was available for 31 patients (Table 1). An example of KRAS G12D detection in plasma DNA by ddPCR is shown in Fig. 2a with the corresponding positive control G12D mutant DNA and WT DNA, as well G12D mutant DNA spiked and non-spiked plasma. 8/31 (26 %) patients were positive for a KRAS mutation. Six patients had the G12D mutation and 1 patient had the G12R and another had the G12V mutation. This included 3 patients with a resectable disease, one with a locally advanced disease and 4 with metastatic disease (Fig. 2b). Seven patients tested for a KRAS mutation had previously received chemotherapy, one was positive for a KRAS mutation and the remaining patients were negative. The concentration of DNA was significantly higher in plasma from patients that tested positive for a mutation in KRAS as compared to those that tested negative (Fig. 2c). Patients that tested positive for a KRAS mutation in plasma had a significantly shorter overall survival than patients that tested negative for a mutation (Fig. 2d), 60 days (95 % CI:19–317) KRAS mutation positive vs 772 days for mutation negative (95 % CI:416–1127) according to the Kaplan Meier analysis (*p =* 0.001). However, due to the small patient cohort we performed a more rigorous statistical analysis of survival in order to confirm this association. The cox regression model (which corrected for the effects of age and sex of patients) showed a significant difference in overall survival for KRAS positive vs KRAS negative patients with a hazard ratio of 12.2 (3.3-45.1, *p =* <0.001) (Additional file 4: Table S2 and Additional file 5: Figure S3). Finally the Weibull regression analysis confirmed these results with a HR 12.2 (3.6-40.7, *p =* <0.001) (Additional file 4: Table S2, Additional file 6: Fig. S4).

### KRAS mutation detection in primary tumor tissue

Paraffin embedded primary tumor tissue was available for 12 of the 31 patients tested for a KRAS mutation in plasma. KRAS mutation detection is summarized in Table 1. 5/12 primary tumors tested wildtype for KRAS and 7/12 tested mutant (4 G12D, 1 G12R, 1 G12V, 1 G12S). KRAS mutation status in primary tissue was available for 5 of the 8 patients that tested positive for KRAS mutation in plasma. The same mutation was confirmed in 3 of these patients whilst the remaining 2 tumor samples tested WT for KRAS. With regard to patients that tested negative for a KRAS mutation in plasma, 4/7 of these patients tested positive for a mutation in the primary tumor (2 G12D, 1 G12R, 1 G12S) and the remaining tested wildtype. Of the 4 that tested negative for a mutation in plasma and positive in the primary tumor, 3 had previously received chemotherapy before sample extraction.

### CTC detection in PDAC patients

**Fig. 3:**
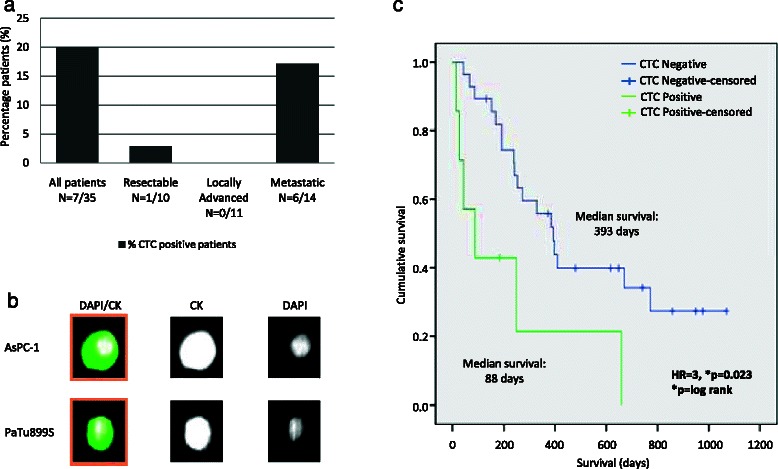
CTC detection whole blood in PDAC cases. **a**. Frequency of CTC in peripheral blood in PDAC. **b**. AsPc-1 and PaTu8988S detection in spiked peripheral blood (100 cells/ml) using the CellSearch® system. **c**. Kaplan Meier survival analysis of CTC status in peripheral blood

CTC data were available for 35 patients (Table 1). CTC were detected in 7/35 (20 %) patients analyzed, this included 6 patients with metastatic disease and 1 patient with a resectable tumor (Fig. 3a). One patient with metastatic disease had 13 CTC, one had 4, another had 5 CTC, one had 1 CTC and two had 3 CTC. The patient with resectable disease had 1 CTC. No CTC were detected in patients with a locally advanced disease. Two patients with CTC determination data had previously received chemotherapy, 1 patient had 5 CTC and the second was negative for CTC. AsPc-1 and PaTu899S pancreatic cancer cell lines were successfully detected by the CellSearch system confirming that tumor cells of pancreatic origin are detectable by this system (Fig. 3b). CTC positive patients had a significantly shorter overall survival (Fig. 3c), 88 days (95 % CI: 27–206) CTC positive vs 393 days CTC negative (95 % CI: 284–501) according to the Kaplan Meier analysis (*p =* 0.0108). A Cox regression analysis with age and sex as cofounders also showed a significant difference in overall survival for CTC positive vs. CTC negative patients with a hazard ratio of 3 (1.16–7.38, p 0.023) (Additional file 4: Table S2 and Additional file 5: Figure S3). A Weibull regression analysis confirmed these results with a HR 2.9(1.16–7.63, *p =* 0.025) (Additional file 4: Table S2, Additional file 6: Fig. S4).

### KRAS mutation detection in CD45 depleted blood samples

**Fig. 4:**
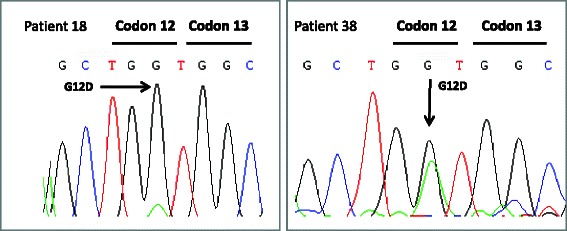
KRAS mutation detection in CD45 depleted blood. The KRAS G12D mutation was detected in 2 patients that tested negative for CTC by the CellSearch® system

CD45 depleted blood samples were available for 9 patients. Exon 2 and 3 of KRAS was successfully PCR amplified in all patients, this included 6 with CTC determinations and 3 without. The G12D mutation was detected in four patients; two of these patients were CTC negative by the CellSearch® system (Fig. 4). Three patients positive for a KRAS G12D mutation in CD45 depleted blood were negative for a KRAS mutation in plasma and another patient negative for a KRAS mutation in depleted blood was positive for the G12D mutation in plasma.

### Mutant KRAS in cfDNA vs CTC detection

Data with regard to both CTC status and KRAS mutation status in plasma was available for 21 patients. 4/5 patients positive for CTC were also positive for a KRAS mutation in plasma. Another patient positive for a G12D mutation in plasma was negative for CTC.

## Discussion

We have demonstrated that tumor specific DNA can be detected in plasma in patients with PDAC. In addition, cfDNA concentration tends to increase with advanced disease stages although this did not correlate with OS. This may be due to the fact that cfDNA concentration is influenced by tumor burden with may be variable among patients due to differences in the clearing of cell debris from the circulation [14].

ddPCR is a sensitive method for the detection of small quantities of DNA and we have demonstrated that as few as 0.5 ng of mutant DNA corresponding to 37 copies can be detected by this technique. However, we did detect some non-specific amplification of G12D mutant DNA with the G12V assay. The specific base affected in these mutations is the same c.35G > A (G12D) and c.35G > T (G12V), thus some non-specific amplification may occur. However, it should be noted that there was no non-specific amplification with WT DNA or G12R mutant DNA (which is affected by a different base c.34G > C).

G12D, G12V and G12R represent the most frequent KRAS mutations found in sporadic PDAC primary tumors with a frequency of 51 %, 29 % and 12 % of all KRAS mutations respectively according to the COSMIC database [17]. However, there are other less frequently occurring mutations such as G12C (2.8 %), G12S (2.2 %), G12A (1.6 %), G13D (0.7 %), Q61H (0.7 % of all primary tumors) that may also be present in cfDNA that have not been tested here, thus the number of KRAS positive patients is probably underestimated. Importantly, we demonstrate that tumor specific DNA can be detected in PDAC plasma, even in patients with a resectable disease that supposedly has not yet metastasized or released CTC into the peripheral blood.

Primary tissue from PDAC patients is limited due to the fact that most patients present with advanced disease and usually only fine-needle aspiration (FNA) biopsies are available. However, we were able to obtain sufficient DNA from 12 of the 31 patients tested for a KRAS mutation in plasma in order to confirm the presence of the same mutation in the primary tumor. The same KRAS mutation found in plasma was also found in the primary tumor in 3 of 5 patients with available tissue. The remaining 2 patients tested WT for KRAS in the primary tumor. This is most likely due to the fact that we performed macro dissection of the tissue in order to obtain tumor DNA and PDAC tumors contain a high proportion of stromal tissue and thus we will ultimately have contaminating non-tumor KRAS WT cells in the sample. Ideally micro-disection of PDAC tissue should be performed to obtain a pure sample of tumor cells, however this was not available in our facility. This combined with the fact that PCR amplification followed by Sanger sequencing is a low sensitivity method for mutation detection, meaning that KRAS mutation detection in these samples is challenging.

Of the 4 patients with mutant KRAS in the primary tumor that were negative for a KRAS mutation in plasma, 3 had previously received chemotherapy. This may have affected the presence of circulating tumor DNA and highlights the importance of sample homogeneity in this type of study and that ideally samples should be extracted prior to starting treatment.

In general the frequency of CTC detection was very low in PDAC cases as compared to other solid tumors such as colorectal cancer where CTC have been detected in 36 % of patients with stage I-IV disease [20] with the CellSearch® system. In addition, the number of CTC detected was very low, we detected a range of 1–13 CTC in patients with metastatic disease as compared to other studies in colorectal cancer where 29 % of patients with stage IV have 3 CTC or more [21], and metastatic prostate and breast cancer where 57 % and 25 % of patients had 5 CTC or more respectively [8, 22]. CTC were most frequently detected in metastatic patients, and one CTC was detected in a patient with resectable disease which falls within the limit of false positive data.

The low detection rate may be due to physiological reasons, such as the fact that pancreatic tumors are generally poorly vascularised and the disease is more localized with metastasis mainly in the liver and peritoneum [23]. However, the low detection rate may also be due to the detection method. The CellSearch® system is based on the detection of cells that express the epithelial markers EpCAM and cytokeratin (CK), thus cells that do not express these antigens will not be detected by this approach. We have shown that cultured cells originating from a pancreatic tumor are successfully identified by the system; however these are adherent cultured cells and thus are likely to express EpCAM at high levels. EpCAM is expressed in many epithelial tumors and thus is a widely used tumor marker. A recent study in a mouse model of PDAC demonstrated that the phenotype of pancreatic circulating epithelial cells is very heterogeneous and only 27 % express EpCAM whereas 40 % express the mesenchymal marker Zeb1 [24]. CTC expressing both epithelial and mesenchymal markers, have been identified in patients with breast and non-small cell lung cancer [25] suggesting that CTC may undergo an epithelial to mesenchymal transition (EMT) and thus exhibit reduced expression of epithelial markers such EpCAM and CK.

These results led us to investigate other methods for the detection of CTC in pancreatic cancer via a marker independent approach. We have shown that negative selection of CD45 expressing cells is a feasible strategy to enrich the CTC population from whole blood. We have demonstrated that patients negative for CTC using the CellSearch® System were positive for a KRAS mutation in CD45 depleted blood indicating that (1) CTC exist in peripheral blood and (2) that there are a sufficient number of cells for detection using this low sensitivity approach, but there is an obvious need to apply the appropriate makers for their detection.

The fact that patients positive for a KRAS mutation in CD45 depleted blood were negative for a KRAS mutation in plasma indicates that the majority of cfDNA is unlikely to come from CTC. This is consistent with previous findings that patients with digestive cancers with detectable cftDNA (circulating free tumor DNA) are not necessarily CTC positive [26].

This pilot study demonstrates that patient’s positive for CTC or KRAS mutations in plasma have a statistically significant poorer overall survival. The liquid biopsy for CTC and cftDNA detection are promising minimally invasive biomarkers in the PDAC setting. However, in order to explore the viability of CTC and cftDNA as prognostic and predictive biomarkers in PDAC we would require serial samples taken during the course of the disease from PDAC cases.

## Conclusions

- KRAS mutant circulating free DNA is a promising marker for the management of patients with PDAC of all stages.
- The concentration of cfDNA may act as a surrogate marker of disease stage, however this needs to be studied in a larger patient cohort.
- CTC detection using the CellSearch® system as a marker in pancreatic cancer is limited due to the low detection rate and the fact that they are usually found in patients with a metastatic disease when treatment options are more limited.
- The CellSearch® system may not be adequate for the detection of CTC in the context of pancreatic cancer. In general the detection of CTC in PDAC is hindered by a lack of data with regard to the phenotype of these cells thus it is difficult to select adequate markers for their detection.

## Additional files

Additional file 1: Table S1. Analysis of clinical parameters in the patient cohorts. (DOC 34 kb)
Additional file 2: Figure S1. Specificity of KRAS mutation assays (G12D, G12R and G12V) determined by ddPCR of KRAS mutant and WT DNA. (PDF 99 kb)
Additional file 3: Figure S2. KRAS G12D mutation detection in spike in plasma samples. (a) G12D mutant DNA detection by ddPCR and (b) correlation of copies of G12D KRAS mutant DNA and spike in concentration. (PDF 45 kb)
Additional file 4: Table S2. Statistical analysis of overall survival with regard to the detection of CTC and mutant KRAS cfDNA in plasma. N.B: The Cox and Weibull regression are corrected by age and sex. (DOC 30 kb)
Additional file 5: Figure S3. Estimated Survival Curves adjusted by sex and age using Cox regression for CTC and KRAS Mutant models. (PDF 23 kb)
Additional file 6: Figure S4. Graphical test of the Weibull assumption. Plot of log(-log(Survival)) vs log(time). When the result is a straight line, survival time is considered to follow a Weibull distribution. (PDF 31 kb)
