# Supplementary material for: Circulating tumor cells (CTC) and KRAS mutant circulating free DNA (cfDNA) detection in peripheral blood as biomarkers in patients diagnosed with exocrine pancreatic cancer
Source: BMC Cancer. 2015 Oct 24;15:797. doi: 10.1186/s12885-015-1779-7 (PMC4619983; doi:10.1186/s12885-015-1779-7)
Supplement: Additional file 1: Table S1. — Analysis of clinical parameters in the patient cohorts. (DOC 34 kb) [file 12885_2015_1779_MOESM1_ESM.doc]

**Supplementary Table 1:** Analysis of clinical parameters in the patient cohorts.

| Variables | | Classification | | |  |
| --- | --- | --- | --- | --- | --- |
|  |  | CTC data only | CTC and KRAS data | KRAS data only | p-value |
| Age | | 70.35(6.15) | 68.28(9.28) | 56.7(11.95) | 0.01613^1^ |
| Survival (days) | | 362.28(296.87) | 395.04(310.19) | 480.9(403.48) | 0.8096^1^ |
| Sex | M | 6 | 10 | 3 | 1^2^ |
|  | F | 8 | 11 | 5 |  |
| Stage | R | 4 | 6 | 4 | 0.9353^2^ |
|  | LA | 5 | 6 | 2 |  |
|  | M | 5 | 9 | 4 |  |
| Survival Analysis Status | Censored | 2 | 9 | 5 | 0.1156^2^ |
|  | Event | 12 | 12 | 5 |  |
