# Supplementary material for: Circulating tumor cells (CTC) and KRAS mutant circulating free DNA (cfDNA) detection in peripheral blood as biomarkers in patients diagnosed with exocrine pancreatic cancer
Source: BMC Cancer. 2015 Oct 24;15:797. doi: 10.1186/s12885-015-1779-7 (PMC4619983; doi:10.1186/s12885-015-1779-7)

Supplementary Figure 1

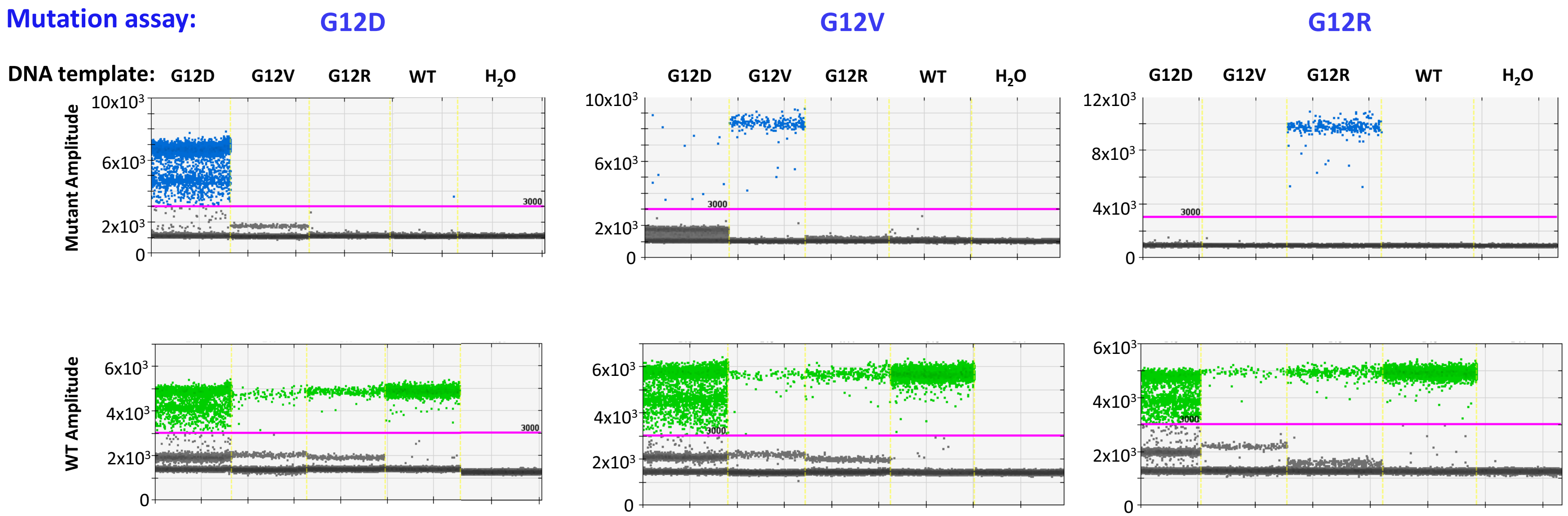

Supplement: Additional file 2: Figure S1. — Specificity of KRAS mutation assays (G12D, G12R and G12V) determined by ddPCR of KRAS mutant and WT DNA. (PDF 99 kb) [file 12885_2015_1779_MOESM2_ESM.pdf]
