# Supplementary figures and images for: Circulating tumor cells (CTC) and KRAS mutant circulating free DNA (cfDNA) detection in peripheral blood as biomarkers in patients diagnosed with exocrine pancreatic cancer
Source: BMC Cancer. 2015 Oct 24;15:797. doi: 10.1186/s12885-015-1779-7 (PMC4619983; doi:10.1186/s12885-015-1779-7)

Supplementary Figure 2

a

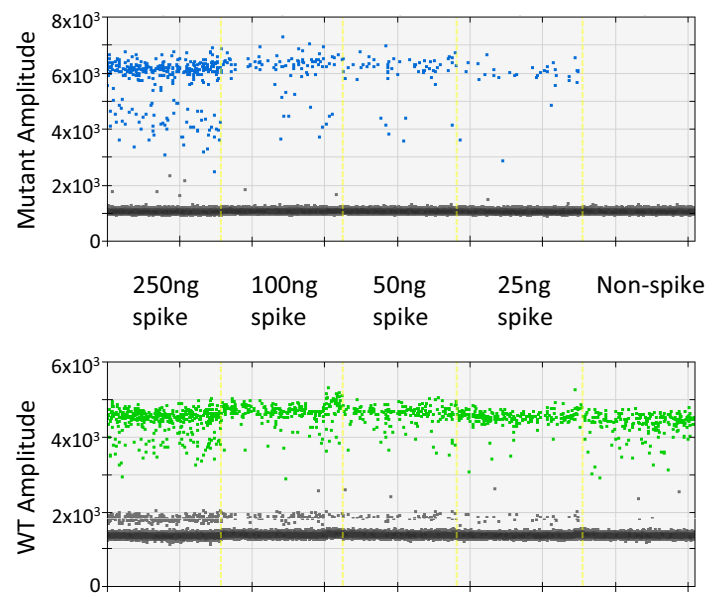

b

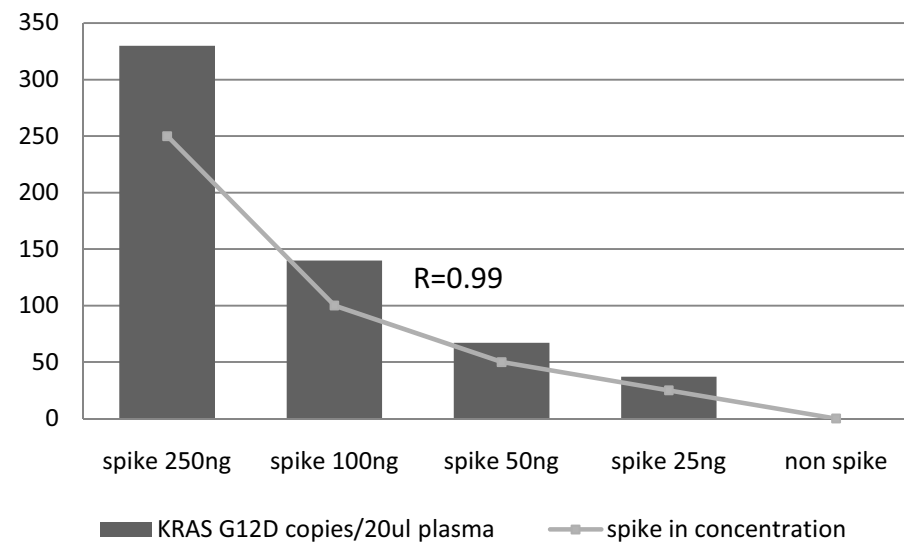

Supplement: Additional file 3: Figure S2. — KRAS G12D mutation detection in spike in plasma samples. (a) G12D mutant DNA detection by ddPCR and (b) correlation of copies of G12D KRAS mutant DNA and spike in concentration. (PDF 45 kb) [file 12885_2015_1779_MOESM3_ESM.pdf]

Supplementary Figure 3

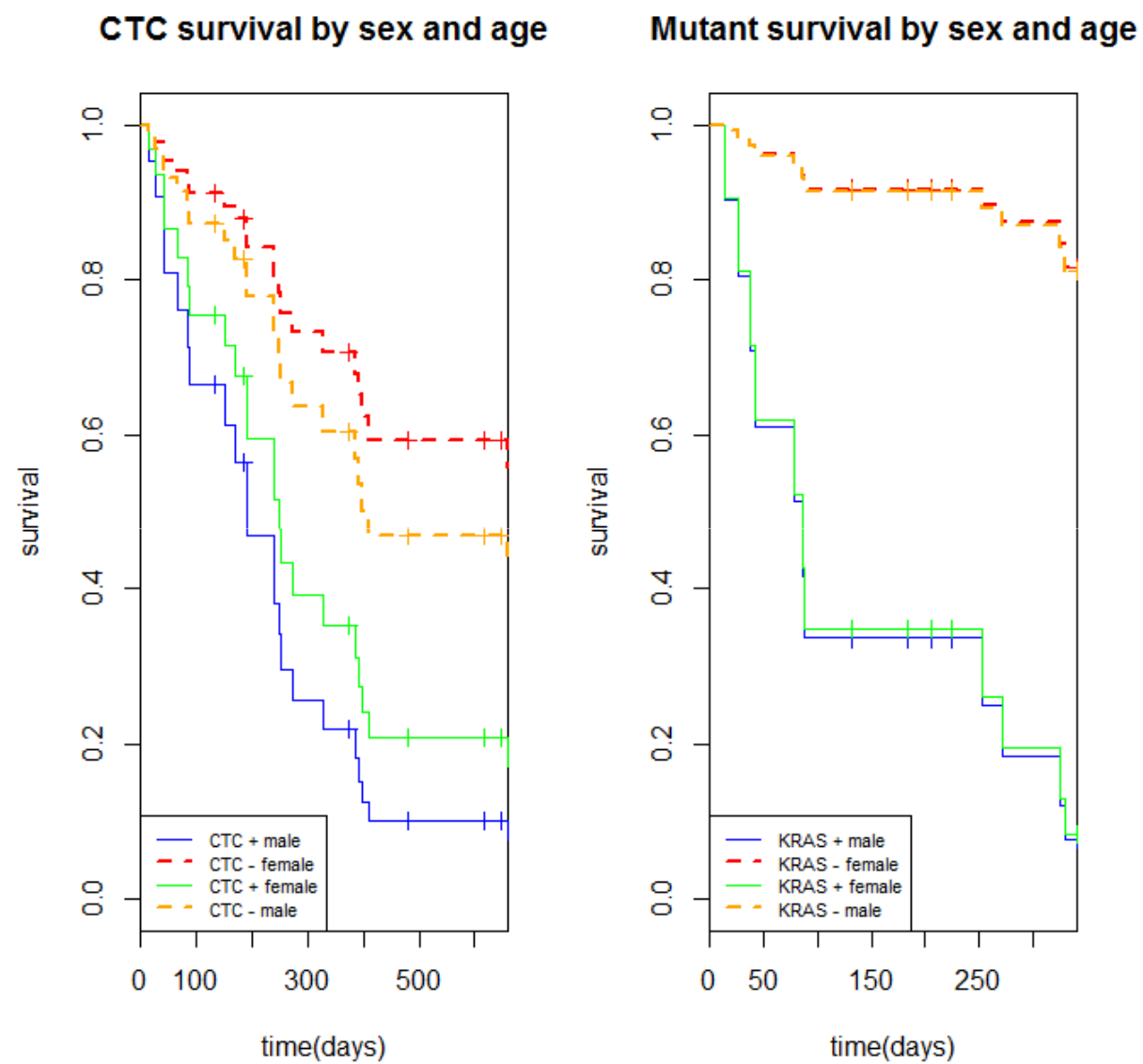

Supplement: Additional file 5: Figure S3. — Estimated Survival Curves adjusted by sex and age using Cox regression for CTC and KRAS Mutant models. (PDF 23 kb) [file 12885_2015_1779_MOESM5_ESM.pdf]

Supplementary Figure 4

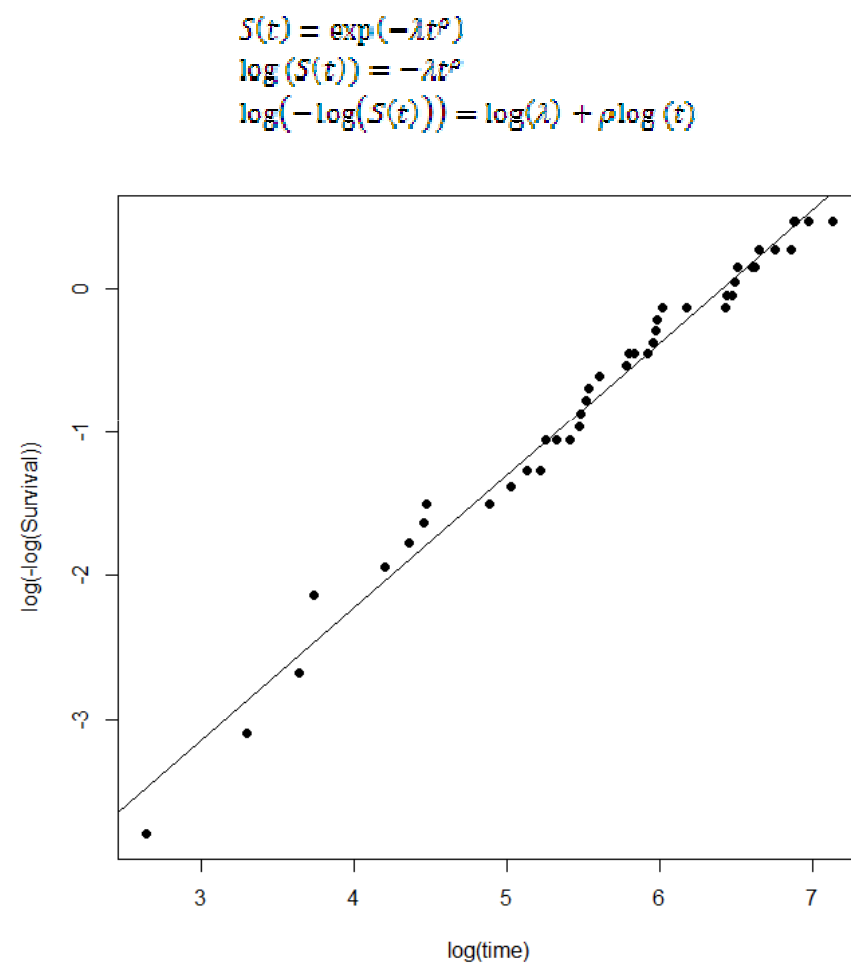

Supplement: Additional file 6: Figure S4. — Graphical test of the Weibull assumption. Plot of log(-log(Survival)) vs log(time). When the result is a straight line, survival time is considered to follow a Weibull distribution. (PDF 31 kb) [file 12885_2015_1779_MOESM6_ESM.pdf]
