# Supplementary material for: Circulating tumor cells (CTC) and KRAS mutant circulating free DNA (cfDNA) detection in peripheral blood as biomarkers in patients diagnosed with exocrine pancreatic cancer
Source: BMC Cancer. 2015 Oct 24;15:797. doi: 10.1186/s12885-015-1779-7 (PMC4619983; doi:10.1186/s12885-015-1779-7)
Supplement: Additional file 4: Table S2. — Statistical analysis of overall survival with regard to the detection of CTC and mutant KRAS cfDNA in plasma. N.B: The Cox and Weibull regression are corrected by age and sex. (DOC 30 kb) [file 12885_2015_1779_MOESM4_ESM.doc]

**Supplementary Table 2:** Statistical analysis of overall survival with regard to the detection of CTC and mutant KRAS cfDNA in plasma.

N.B: The Cox and Weibull regression are corrected by age and sex.

|  |  | **p-value** | **Regression Coefficient** | **HR** |
| --- | --- | --- | --- | --- |
| **Kaplan Meier** | CTC | 0.0108 |  |  |
|  | KRAS | <0.001 |  |  |
| **Cox regression** | CTC | 0.023 | 1.1018± 0.4849 | 3.0095 (1.163, 7.785) |
|  | KRAS | 0.00018 | 2.502 ± 0.6676 | 12.207(3.298,45.175) |
| **Weibull regression** | CTC | 0.0248 | -1.0270 ± 0.4575 | 2.979 (1.162 ,7.637) |
|  | KRAS | <0.001 | -2.153 ± 0.479 | 12.228(3.673, 40.713) |
